# MicroRNA-125b is a key epigenetic regulatory factor that promotes nuclear transfer reprogramming

**DOI:** 10.1074/jbc.M117.796771

**Published:** 2017-08-09

**Authors:** Jingcheng Zhang, Pengxiang Qu, Chuan Zhou, Xin Liu, Xiaonan Ma, Mengyun Wang, Yongsheng Wang, Jianmin Su, Jun Liu, Yong Zhang

**Affiliations:** From the Key Laboratory of Animal Biotechnology of the Ministry of Agriculture, College of Veterinary Medicine, Northwest A&F University, Yangling 712100, Shaanxi, China

**Keywords:** cloning, embryo, histone methylation, microRNA (miRNA), reprogramming

## Abstract

Somatic cell nuclear transfer (SCNT)-mediated reprogramming is a rapid, efficient, and sophisticated process that reprograms differentiated somatic cells to a pluripotent state. However, many factors in this elaborate reprogramming process remain largely unknown. Here, we report that the microRNA (miR) miR-125b is an important component of SCNT-mediated reprogramming. Luciferase reporter assay, quantitative PCR, and Western blotting demonstrated that miR-125b directly binds the 3′-untranslated region of SUV39H1, encoding the histone-lysine *N*-methyltransferase SUV39H1, to down-regulate histone H3 lysine-9 tri-methylation (H3K9me3) in SCNT embryos. Furthermore, the miR-125b/SUV39H1 interaction induced loss of SUV39H1-mediated H3K9me3, caused heterochromatin relaxation, and promoted the development of SCNT embryos. Transcriptome analyses of SCNT blastomeres indicated that HNF1 homeobox B (HNF1B), a gene encoding a transcription factor downstream of and controlled by the miR-125b/SUV39H1 axis, is important for conferring developmental competence on preimplantation embryos. We conclude that miR-125b promotes SCNT-mediated nuclear reprogramming by targeting SUV39H1 to decrease the deposition of repressive H3K9me3 modifications.

## Introduction

Somatic cell nuclear transfer (SCNT)^4^-mediated reprogramming is a rapid and efficient method used to reprogram differentiated somatic cells to a pluripotent state (1). However, SCNT embryonic development is a very complex process in which all stages are strictly controlled, including protein exchange between the donor nucleus and ooplasm, donor nuclear chromatin remodeling, and embryo gene transcriptional activation (2–4). Each stage is spatially and temporally specific and dependent on precise intrinsic and extrinsic signal regulation. In particular, transcriptional regulation is an essential element in which microRNA (miRNA) is likely to play an important role as a post-transcriptional regulator during nuclear reprogramming and embryonic development (5, 6).

By definition, miRNAs are 21–23-nucleotide-long noncoding RNAs that base pair with the 3′-untranslated regions (UTRs) of target mRNAs to induce degradation and prevent translation (7–9). These important regulatory molecules therefore influence a myriad of cell functions, including the generation of induced pluripotent stem cells and regulation of embryogenesis and animal reproduction (5, 10, 11). However, limited information is available regarding the miRNA(s) responsible for successful nuclear transfer reprogramming.

The recently established miRNA expression profiles of mammalian gonads (6, 10) demonstrate that miR-125b is one of the most abundant miRNAs detected in early embryos (6, 10). However, little is known about the functions and mechanisms of miR-125b in embryonic development, especially during nuclear reprogramming. Recently, *RYBP* (12), a direct target of miR-125b, was found to suppress gene activation within the preimplantation and embryonic genome in early embryonic cells (13). These results suggest that miR-125b may play a potentially important role in SCNT-mediated reprogramming.

Epigenetic modifications work in concert with genetic mechanisms to regulate transcriptional activity during nuclear reprogramming and embryonic development (14). Successful SCNT relies on the erasure of epigenetic modifications from highly differentiated somatic cells followed by the temporally appropriate reestablishment of new epigenetic modifications similar to those found in embryonic cells (15). Numerous experiments have shown incomplete and inappropriate epigenetic reprogramming from SCNT, and developmental abnormalities observed in unsuccessful SCNT embryos have largely been attributed to these aberrant modifications (16), including the trimethylation of histone tails at lysine 9 (H3K9me3) and lysine 27 (H3K27me3) (15, 17–19). H3K9me3 and H3K27me3, which have been associated with chromatin compaction and transcription suppression, dynamically and inappropriately modulate the chromatin structure and function and contribute to abnormal gene expression (17).

In this report we found that miR-125b/SUV39H1 axis promotes embryonically expressed gene expression by inducing SUV39H1-methylated H3K9me3 loss and facilitates SCNT reprogramming. This finding indicates that miR-125b/SUV39H1-dependent histone modification losses are responsible for embryo gene activation and SCNT-mediated nuclear reprogramming.

## Results

### miR-125b is strongly expressed in SCNT embryos and regulates the reprogramming capacity

A previous study has revealed that miR-125b transcription predominantly occurred in bovine oocytes (20), and we demonstrated strong miR-125b expression in mature oocytes and early embryos by quantitative real-time PCR (qPCR; supplemental Fig. S1). Compared with IVF embryos, more abundant miR-125b was observed in SCNT embryos during the early stage (supplemental Fig. S1). Therefore, we hypothesized that miR-125b might be important for successful SCNT-mediated nuclear reprogramming.

**Figure 1.**
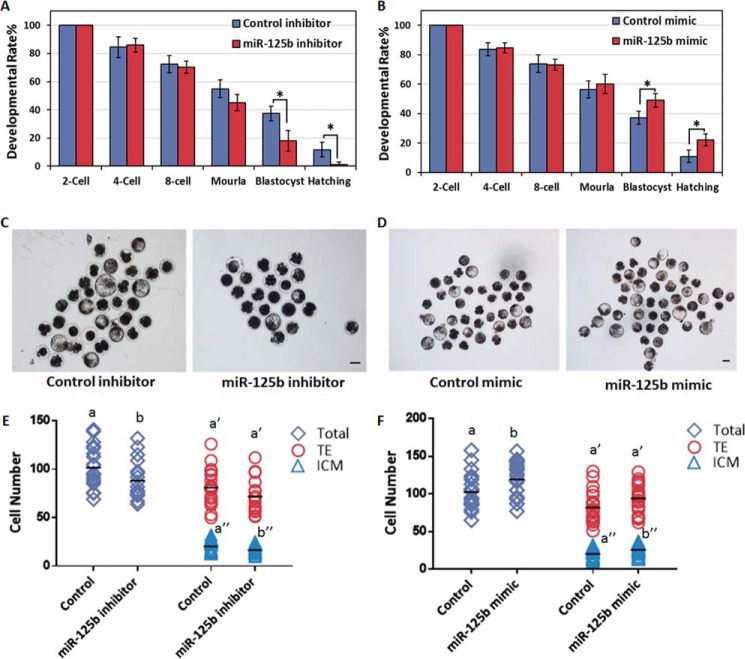
**miR-125b regulates NT-mediated nuclear reprogramming.** *A* and *B*, miR-125b regulates the formation and hatching of NT-blastocysts. Shown are control and miR-125b inhibitor (*A*)- or miR-125b mimic-injected embryos (*B*). Data indicate the percentages of embryos that reached the indicated stages (*, *p* < 0.05). *Error bars* indicate S.D. *C* and *D*, representative images of control, miR-125b inhibitor (*C*) and miR-125b mimic-injected somatic cell NT-embryos (*D*) at 8 days post-activation. *Scale bar* = 150 μm. *E* and *F*, plots show the total: ICM and TE cell numbers of control; miR-125b inhibitor (*E*) and miR-125b mimic-injected somatic cell NT-embryos (*F*). Different characters *a*, *b* (total), a′, *b*′ (TE), or *a*″, *b*″ (ICM) above the plots indicate significant differences (*p* < 0.05).

To test this hypothesis, we first injected reconstructed SCNT embryos (2–3 h post-activation; *i.e.* one-cell stage) with either a miR-125b inhibitor or mimic. Then, we next tracked the *in vitro* developmental capacities of SCNT embryos as an indicator of reprogramming efficiency. Although injection with miR-125b inhibitor did not show an obvious effect on the developmental rates of most SCNT embryos before the end of the eight-cell stage and completion of embryo gene activation (EGA), developmental retardation became evident during the blastocyst stage (Fig. 1, *A* and *C*, and Table 1), and the hatching rates and quality of the blastocysts were largely reduced (Fig. 1, *A* and *E*, Table 1, and supplemental Fig. S2*A*). Furthermore, as expected, the overexpression of miR-125b (using an injected mimic) had no effect on the developmental rate of SCNT embryos before the morula stage but led to an increased rate during the blastocyst stage (Fig. 1, *B* and *D*, and Table 1). More importantly, the hatching rates and blastocyst quality improved after miR-125b overexpression (Fig. 1, *B* and *F*, Table 1, and supplemental Fig. S2*B*). This finding suggests that miR-125b alters the developmental capacities of SCNT embryos, especially after EGA, and is important for SCNT-mediated nuclear reprogramming.

**Table 1:** **Effect of miR-125b on *in vitro* development of NT-embryos** Values with different superscripts within columns denote significant difference (*p* < 0.05).

Groups	Repeats	Reconstructed embryos	% cleaved per 1-cell ± S.D.	% 4-cell per 2-cell ± S.D.	% 8-cell per 2-cell ± S.D.	% morula per 2-cell ± S.D.	% blast per 2-cell ± S.D.	% hatching per 2-cell ± S.D.
Control inhibitor	3	91	86.7 ± 6.0	84.5 ± 7.3	72.4 ± 6.3	54.9 ± 6.3	37.4 ± 5.3^a^	11.8 ± 5.3^a^
miR-125b inhibitor	3	94	89.6 ± 11.0	85.9 ± 4.8	70.2 ± 4.3	45.1 ± 5.8	18.1 ± 7.4^b^	1.2 ± 2.0^b^
Control mimic	3	114	86.9 ± 4.2	83.8 ± 4.5	73.9 ± 5.9	56.4 ± 5.8	37.2 ± 4.3^a^	11.0 ± 4.4^a^
miR-125b mimic	3	126	84.4 ± 6.5	84.7 ± 3.3	73.2 ± 3.7	60.1 ± 6.6	49.1 ± 4.7^b^	22.3 ± 4.2^b^

### miR-125b triggers histone modifications, including a decrease in H3K9me3 deposition and heterochromatin relaxation

**Figure 2.**
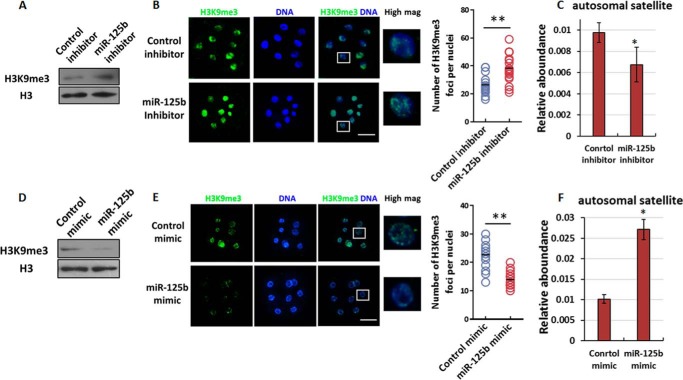
**Effect of miR-125b on H3K9me3 levels and heterochromatin deposition.** *A*, immunoblotting analysis of the H3K9me3 abundance in SCNT embryos after miR-125b knockdown. The protein expression levels are normalized to histone H3 levels. *B*, immunofluorescence analysis of H3K9me3 in miR-125b-knockdown SCNT embryos at the eight-cell stage. *Scale bars* = 100 μm. The *graph at right* shows the number of heterochromatin foci per nuclei (**, *p* < 0.01). *C*, qPCR analysis of satellite expression in SCNT embryos after miR-125b knockdown. The GAPDH expression level was set to 1.0. The results were obtained from triplicate experiments. Each experiment used 10–15 embryos (*, *p* < 0.05). *Bars* indicate the S.E. *D* and *E*, immunoblotting (*D*) and immunofluorescence (*E*) analyses of H3K9me3 levels in miR-125b-overexpressing SCNT embryos, as described in *A* and *B. F*, qPCR analysis of satellite expression in miR-125b-overexpressing SCNT embryos at the eight-cell stage (*, *p* < 0.05). The GAPDH level was set to 1.0. *Bars* indicate the S.E.

Recently, several groups independently demonstrated that aberrant histone modifications lead to nuclear reprogramming failures (19, 21–24). In accordance with the finding that miR-125b affects EGA completion in SCNT embryos, we wondered whether this effect on reprograming might be mediated by altered histone modification. Thus, we examined global H3 methylation and observed a global increase in H3K9me3 during the EGA stage (eight-cell stage) after miR-125b inhibitor injection (Fig. 2*A*). Immunostaining revealed an increased number of H3K9me3 foci in miR-125b inhibitor-injected embryos (Fig. 2*B*), the heterochromatin is enriched for H3K9me3 in mammalian cells, we hypothesized that a miR-125b inhibitor affects heterochromatin structure by inducing H3K9me3 accumulation. To confirm this hypothesis, we selectively evaluated the transcripts of several genomic repetitive elements that serve as markers of altered heterochromatin structure (25). Specifically, the expression of these transcripts increases upon heterochromatin relaxation (25, 26). We observed a block in the relative expression of satellites at the eight-cell stage when miR-125b was inhibited (Fig. 2*C*), suggesting less relaxation of the heterochromatin structure. Concomitantly, miR-125b overexpression led to clear reduces in global H3K9me3 levels and decreased the number of H3K9me3 foci among the heterochromatin (Fig. 2, *D–E*). Furthermore, satellite expression was enhanced in SCNT embryos injected with miR-125b mimics (Fig. 2*F*). Taken together, these findings indicate that miR-125b reduces H3K9me3 and promotes heterochromatin relaxation in SCNT embryos.

### miR-125b directly targets SUV39H1

**Figure 3.**
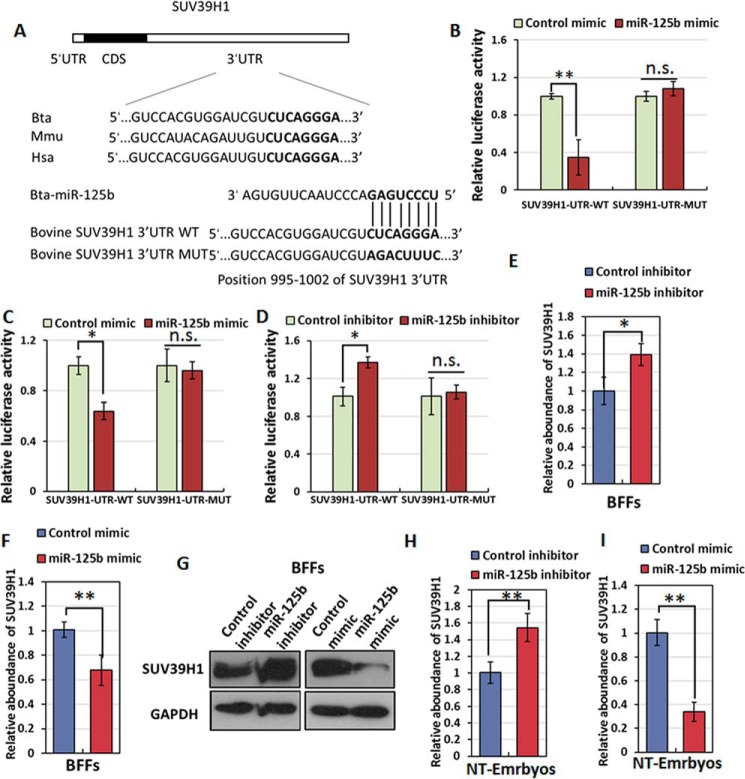
**SUV39H1 is a direct target of miR-125b.** *A*, putative miR-125b target binding sites in the bovine *SUV39H1* UTRs. *CDS*, coding sequence. *B*, relative luciferase activity in HEK293T cells co-transfected with miR-125b and reporter constructs containing the mutant *SUV39H1* 3′-UTR. *n.s.*, not significant. *C* and *D*, relative luciferase activities in BFFs co-transfected with a reporter construct containing the mutant *SUV39H1* 3′-UTR and either a miR-125b (*C*) or miR-125b inhibitor (*D*). *E* and *F*, the relative SUV39H1 mRNA levels in BFFs were determined by qPCR 2 days after miR-125b inhibitor transfection (*E*) or miR-125b overexpression (*F*). *Bars* indicate the S.E. *G*, relative SUV39H1 protein levels in BFFs were determined by immunoblotting 2 days after miR-125b overexpression or miR-125b inhibitor transfection. *H* and *I*, relative SUV39H1 mRNA levels in four-cell stage SCNT embryos were determined by qPCR after miR-125b inhibitor (*H*) or miR-125b injection (*I*). *Bars* indicate the S.E. *, *p* < 0.05; **, *p* < 0.01.

We next addressed the molecular mechanism by which miR-125b regulates nuclear reprogramming during SCNT embryonic development. Individual miRNAs can regulate target mRNAs at the post-transcriptional level to inhibit target gene expression through translational repression or by directly degrading the nucleic acid (9). We accordingly searched for potential mRNA targets of miR-125b using the PicTar, miRanda, and TargetScan algorithms. Among the predicted candidates, the 3′-UTR of *SUV39H1*, which encodes a protein important for both histone modification and embryonic development, contains a miR-125b target-binding site (Fig. 3*A*) (21, 27).

To directly test whether *SUV39H1* is a target of miR-125b, the 3′-UTR of *SUV39H1* was cloned in a luciferase reporter vector, and the resulting construct was co-transfected with a miR-125b mimic into the human cell line HEK293T. Luciferase expression effectively decreased after the co-expression of miR-125b with the *SUV39H1* 3′-UTR (Fig. 3*B*). To test whether the predicted miR-125b target site in the *SUV39H1* 3′-UTR is critical for the miR-125b-mediated repression of SUV39H1 expression, we introduced mutations into the seed sequences of the predicted miR-125b-binding sites (Fig. 3*A*). These mutations within the *SUV39H1* 3′-UTR abolished the inhibitory effects of miR-125b on luciferase expression (Fig. 3*B*), suggesting that SUV39H1 is directly regulated by miR-125b. We also tested whether miR-125b regulates SUV39H1 expression levels in bovine cells and embryos and observed similar results from a luciferase reporter assay conducted in bovine fetal fibroblast cells (BFFs) (Fig. 3*C*). Additionally, we conducted a luciferase reporter assay in the presence of a miR-125b inhibitor to exclude the effects of endogenous miR-125b on luciferase activity and observed a much higher luciferase activity level from the *SUV39H1* 3′-UTR construct in the presence of miR-125b inhibitors compared with the control (Fig. 3*D*).

To further confirm the regulatory relationship between miR-125b and SUV39H1, we performed qPCR and immunoblot assays to determine the SUV39H1 mRNA and protein levels in BFFs. We further determined that the endogenous SUV39H1 level was suppressed in response to miR-125b overexpression and enhanced in response to miR-125b inhibition in both BFFs and SCNT embryos. (Fig. 3, *E–I*). Collectively, these results demonstrate that miR-125b negatively regulates SUV39H1 expression at the post-transcriptional level in BFFs and SCNT embryos.

### miR-125b promotes SCNT-mediated reprogramming by causing a loss of SUV39H1-mediated H3K9me3 and inducing heterochromatin structural changes

**Figure 4.**
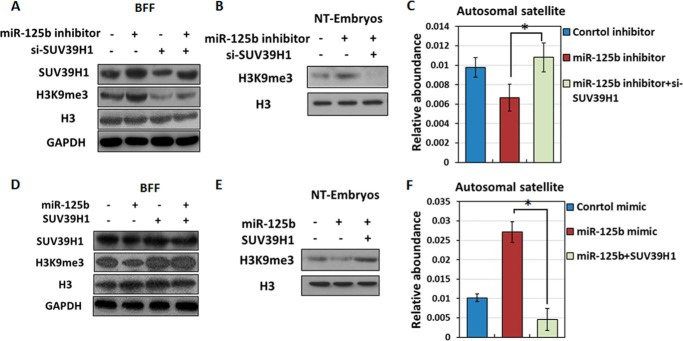
**Effect of the miR-125b/SUV39H1 axis on H3K9me3 levels and heterochromatin deposition.** *A* and *B*, immunoblotting analysis of H3K9me3 levels after knockdown of both miR-125b and SUV39H1 in BFFs (*A*) or SCNT embryos (*B*). Protein expression levels were normalized to histone H3 levels. *C*, qPCR analysis of satellite expression in SCNT embryos after knockdown of both miR-125b and SUV39H1 (*, *p* < 0.05). The GAPDH level was set to 1.0. *Bars* indicate the S.E. *D* and *E*, immunoblotting analysis of H3K9me3 levels after overexpressing both miR-125b and SUV39H1 in BFFs (*D*) and SCNT embryos (*E*). Protein expression levels were normalized to the histone H3 levels. *F*, qPCR analysis of satellite expression in SCNT embryos after overexpressing both miR-125b and SUV39H1 (*, *p* < 0.05). The GAPDH level was set to 1.0. *Bars* indicate the S.E.

As demonstrated above, miR-125b mediates the loss of H3K9me3 and promotes heterochromatin relaxation in SCNT embryos during EGA stage (eight-cell stage) and also negatively regulates the abundance of the histone methyltransferase SUV39H1. Accordingly, we investigated whether miR-125b affects H3K9me3 levels via SUV39H1. After confirming knockdown efficiency (supplemental Fig. S3), we performed immunoblot analysis which revealed that SUV39H1-knockdown BFFs and eight-cell-stage SCNT embryos did not exhibit an increase in H3K9me3 in response to miR-125b inhibitor treatment (Fig. 4, *A–B*). In SCNT embryos, SUV39H1 knockdown also activated satellite expression (Fig. 4*C*). Therefore, SUV39H1 down-regulation endowed SCNT embryos with resistance to miR-125b inhibitor-induced H3K9me3 and heterochromatin accumulation in embryos.

We also evaluated the role of SUV39H1 overexpression in BFFs and EGA-stage SCNT embryos through co-transfer experiments with miR-125b mimics. Unsurprisingly, SUV39H1-overexpressing BFFs and SCNT embryos could resist the miR-125b mimic-induced decrease in H3K9me3 (Fig. 4, *D* and *E*). Additionally, SUV39H1 overexpression repressed satellite expression in eight-cell SCNT embryos (Fig. 4*F*). These findings, which are consistent with the results obtained from the miRNA inhibitor/siRNA-injected group, suggest that the miR-125b/SUV39H1 axis induces the loss of SUV39H1-mediated H3K9me3 and structural changes in heterochromatin.

**Figure 5.**
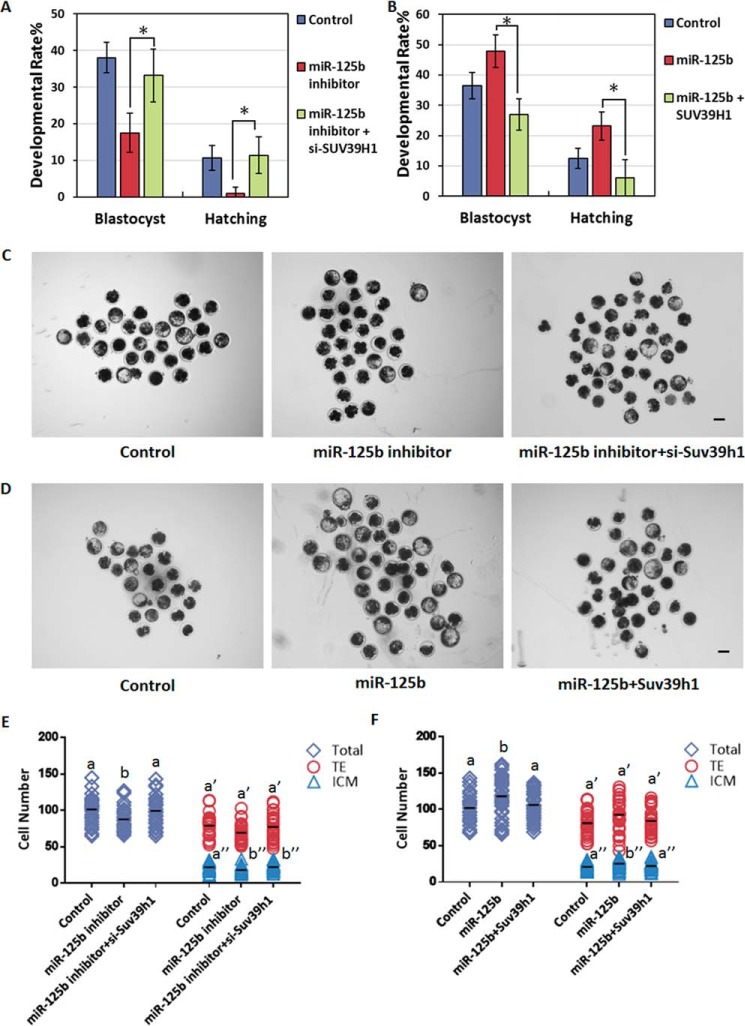
**The miR-125b/SUV39H1 axis regulates development of NT embryos.** *A* and *B*, the miR-125b/SUV39H1 axis regulates NT-blastocyst formation and hatching. Shown are control embryos and the embryos injected with either a miR-125b inhibitor and si-SUV39H1 (*A*) or a miR-125b mimic and SUV39H1 (*B*). The percentages of embryos that reached the indicated stages are shown (*, *p* < 0.05). *Error bars* indicate S.D. *C* and *D*, representative images of control SCNT embryos and those injected with either a miR-125b inhibitor and si-SUV39H1 (*C*) or a miR-125b mimic and SUV39H1 (*D*) at 8 days post-activation. *Scale bar* = 150 μm. *E* and *F*, plots show the total: ICM and TE cell numbers of control; miR-125b inhibitor (*E*) and miR-125b mimic-injected somatic cell NT-embryos (*F*). Different characters *a*, *b* (total), a′, b′ (TE), or a″, b″ (ICM) above plots indicate significant differences (*p* < 0.05).

miR-125b is critically important for SCNT embryonic development, especially after EGA. The above findings suggest that the effects of the miR-125b/SUV39H1 axis on histone modification and chromatin structure might alter EGA gene expression and thereby affect nuclear reprogramming in SCNT embryos. To test the effect on SCNT embryo capacity, we co-injected pronuclear-stage SCNT embryos with a miR-125b inhibitor and either siRNAs targeting SUV39H1 or control siRNAs and tracked preimplantation development to assess the nuclear reprogramming efficiency. SUV39H1 knockdown blocked the miR-125b inhibitor-induced increases in H3K9me3 deposition and heterochromatin formation (Fig. 4, *B* and *C*) and rescued the developmental defects of SCNT embryos caused by miR-125b depletion (Fig. 5, *A*, *C*, and *E*, Table 2, and supplemental Fig. S2*C*). In contrast, SUV39H1 overexpression antagonized the loss of H3K9me3, promoted by the miR-125b mimic, and promoted heterochromatin formation (Fig. 4, *E* and *F*), whereas suppressed facilitation of SCNT embryos caused by miR-125b (Fig. 5, *B*, *D*, and *F*, Table 2, and supplemental Fig. S2*D*). These results suggest that down-regulation of SUV39H1, which reduces H3K9me3 and accelerates heterochromatin relaxation in EGA stage SCNT embryos, is important for miR-125b-induced reprogramming.

**Table 2:** **Effect of miR-125b/SUV39H1 axis on *in vitro* development of NT-embryos** Values with different superscripts within columns denote significant difference (*p* < 0.05).

Groups	Repeats	No. of reconstructed 1-cell embryos	% cleaved per 1-cell ± S.D.	% blast per 2-cell ± S.D.	% hatching per 2-cell ± S.D.
Control	3	97	86.6 ± 3.3	38.0 ± 4.3^a^	10.7 ± 3.4^a^
miR-125b inhibitor	3	99	88.6 ± 5.2	17.5 ± 5.3^b^	1.0 ± 1.7^b^
miR-125b inhibitor + siSUV39H1	3	107	82.4 ± 3.6	33.2 ± 7.2^a^	11.4 ± 5.0^a^
Control	3	92	85.8 ± 6.7	36.5 ± 4.3^a^	12.4 ± 3.3^a^
miR-125b	3	119	84.7 ± 2.1	47.9 ± 5.4^b^	23.2 ± 4.7^b^
miR-125b+SUV39H1	3	95	85.3 ± 3.6	27.0 ± 5.1^a^	6.0 ± 6.0^a^

### The miR-125b/SUV39H1 axis promotes EGA and alters downstream gene HNF1B expression

**Figure 6.**
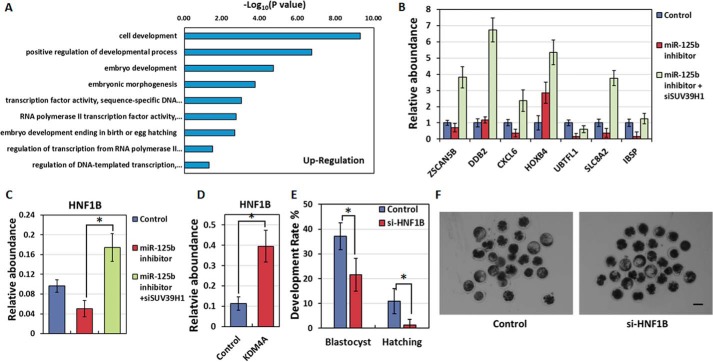
**Identification of genes downstream of the miR-125b/SUV39H1 axis.** *A*, Gene Ontology analysis of up-regulated genes in SUV39H1-knockdown SCNT embryos. *B*, qPCR analysis of EGA-related genes in eight-cell SCNT embryos. *C*, qPCR analysis of HNF1B expression levels in control 8-cell SCNT embryos and those injected with miR-125b inhibitor in the presence or absence of SUV39H1 (*, *p* < 0.05). The GAPDH level was set to 1.0. Results were obtained from triplicate experiments. *Bars* indicate the S.E. *D*, qPCR analysis of gene expression levels in control and KDM4A-injected eight-cell SCNT embryos (*, *p* < 0.05). The GAPDH level was set to 1.0. Results were obtained from triplicate experiments. *Bars* indicate the S.E. *E*, quantification of embryos that developed to the blastocyst stage after injection with control siRNA or si-HNF1B. The percentages of embryos that reached the indicated stages are shown (*, *p* < 0.05). The *error bars* indicate S.D. *F*, representative images of control and si-HNF1B injected SCNT embryos at 8 days post-activation. *Scale bar* = 150 μm.

To determine how the miR-125b/SUV39H1/H3K9me3 axis affects SCNT reprogramming, especially EGA, we analyzed the gene expression profiles of SUV39H1-knockdown eight-cell nuclear transfer (NT)-embryos and identified 3144 up-regulated genes compared with the control. A Gene Ontology (GO) analysis of these genes identified the activation of “embryo development,” “RNA polymerase II transcription factor activity,” and “transcription factor activity, sequence-specific DNA binding” (Fig. 6*A*), indicating that SUV39H1 knockdown promotes EGA and embryonic development during NT-mediated nuclear reprogramming.

We next attempted to detect the key genes controlled by miR-125b/SUV39H1/H3K9me3 axis in each of these GO categories and found that the miR-125b/SUV39H1 axis could promote EGA, as demonstrated by the expression of several EGA markers (Fig. 6*B*). Furthermore, through microinjection and qPCR experiments, we confirmed that *HNF1B*, a gene classified in both the RNA polymerase II transcription factor activity and transcription factor activity, sequence-specific DNA binding categories, was affected by the miR-125b/SUV39H1 axis (Fig. 6*C*). To further confirm that *HNF1B* is regulated by H3K9me3, we injected KDM4A, a specific H3K9me3 demethylase, and observed significantly enhanced *HNF1B* expression compared with the control (Fig. 6*D*). These results demonstrate that *HNF1B* is controlled by the miR-125b/SUV39H1/H3K9me3 pathway.

*HNF1B*-null mouse embryos exhibited an absence of blastocoels and implantation lethality, thus suggesting the importance of this gene for NT-mediated nuclear reprogramming (28, 29). The miR-125b/SUV39H1/H3K9me3 axis might improve the reprogramming capacities of early embryos by inducing *HNF1B* expression and thereby increasing mRNA transcription. Indeed, *HNF1B* depletion reduced the developmental competence of bovine SCNT embryos (Fig. 6, *E* and *F*, Table 3). Taken together, these data indicate that the miR-125b/SUV39H1 axis affects EGA within SCNT embryos. Furthermore, *HNF1B* expression, which is regulated by the miR-125b/SUV39H1/H3K9me3 axis, is a critical step in the developmental competence of bovine SCNT embryos.

**Table 3:** **Effect of HNF1B on *in vitro* development of NT-embryos** Values with different superscripts within columns denote significant difference (*p* < 0.05).

Groups	Repeats	No. of reconstructed 1-cell embryos	% cleaved per 1-cell ± S.D.	% blast per 2-cell ± S.D.	% hatching per 2-cell ± S.D.
Control	3	90	89.1 ± 3.7	34.1 ± 5.4^a^	10.9 ± 5.0^a^
si-HNF1B	3	99	87.9 ± 6.1	21.5 ± 6.6^b^	1.3 ± 2.2^b^

## Discussion

As described earlier, SCNT is a rapid but complex process that can be used to reprogram a differentiated somatic cell into a pluripotent state (30). Therefore, identification and characterization of the regulator of this intricate process can help us to further understand the NT-mediated nuclear reprogramming. Through the present study, we found that the miR-125b/SUV39H1 axis is important for the SCNT-induced reprogramming by reducing H3K9me3 deposition, promoting heterochromatin relaxation, and embryo gene activation.

A previous study observed strong miR-125b expression in oocytes and preimplantation embryos (6), and miR-125b overexpression in GV oocytes was found to inhibit the development of fertilized eggs by targeting LIN28A/Seabox (31). However, in our model, although we demonstrated strong miR-125b expression before the eight-cell stage, LIN28A/Seabox is expressed at very low levels in bovine preimplantation embryos (supplemental Fig. S4*A*). Furthermore, during EGA, genes downstream of miR-125b-LIN28A/SEABOX were not affected by miR-125b inhibitors (supplemental Fig. S4*B*), suggesting that the miR-125b-LIN28A/SEABOX pathway does not exist in bovine SCNT embryos and that miR-125b targets other genes. Accordingly, we found that SUV39H1, which encodes a protein involved in histone methylation, nuclear reprogramming, and embryonic development, was down-regulated by miR-125b in SCNT embryos. By measuring satellite expression, we further demonstrated that the miR-125b/SUV39H1 axis could induce a SUV39H1-mediated loss of H3K9me3 and thus promote structural changes in heterochromatin, consistent with previous reports of the miR-125b/SUV39H1 axis effects on hematopoietic stem cells (32). In addition, several studies demonstrated that aberrant histone modifications and heterochromatin deposition lead to post-EGA reprogramming failures (15, 17, 21). Thus, we suppose that the miR-125b-mediated inhibition of SUV39H1 might facilitate NT reprogramming. To test this point, we blocked the effect of miR-125b inhibition in SCNT embryos by using si-SUV39H1 and observed a reversal of developmental failure; in contrast, SUV39H1 overexpression reversed the promotion of SCNT embryos caused by miR-125b. We therefore conclude that SUV39H1 is important for miR-125b-induced SCNT reprogramming because of its abilities to reduce the H3K9me3 level and alter heterochromatin distribution.

Recent studies that reported the effects of H3K9me3 on SCNT used an additional histone demethylase to remove H3K9me3 from reprograming-resistant regions and to significantly improve the reprogramming efficiency of SCNT (17, 21, 24). We selected KDM4B, a H3K9me3 demethylase that is highly expressed during the early SCNT embryos (supplemental Fig. S5), to compare the positive effects of miR-125b and histone H3K9me3 demethylase. Similar to miR-125b, KDM4B also improved the blastocyst formation rates (supplemental Fig. S6 and Table 4), but the level of improvement was not as drastic as that of mice. One possible explanation for this difference is the relatively imperfect bovine *in vitro* culture system. In bovine, only a small part of cleaved IVF embryo has the capacity to develop into the blastocyst stage (>90% in mice *versus* 46–50% in bovine) (33–37).

**Table 4:** **Preimplantation development of SCNT embryos injected with KDM4B** Values with different superscripts within columns denote significant difference (*p* < 0.05).

Groups	Repeats	Reconstructed embryos	% cleaved per 1-cell ± S.D.	% 4-cell per 2-cell ± S.D.	% 8-cell per 2-cell ± S.D.	% morula per 2-cell ± S.D.	% blast per 2-cell ± S.D.	% hatching per 2-cell ± S.D.
Control	3	94	87.1 ± 8.0	84.3 ± 7.8	73.4 ± 6.8	56.0 ± 6.5	36.4 ± 4.3^a^	12.3 ± 4.8^a^
KDM4B	3	102	87.5 ± 5.2	86.1 ± 6.0	76.0 ± 6.4	63.4 ± 5.9	53.4 ± 6.9^b^	24.4 ± 3.9^b^

In mammals, H3K9me3 affects transcription silencing and heterochromatin formation (38). Because the nuclear lamina can interact with and tether heterochromatin, thereby reducing access to gene silencing abilities (39), H3K9me3-initiated heterochromatin structural changes can obstruct transcriptional activation. In this study, we used RNA-seq and qPCR analyses to confirm that the miR-125b/SUV39H1 axis could alter EGA. In addition to SUV39H1, other miR-125b target genes, including RYBP and ACVR2A/SMAD2, can regulate EGA or reprogramming. However, this topic will require elucidation in future studies. Moreover, our comparison of the transcriptomes of wild-type and si-SUV39H1 SCNT embryos led to the identification of several downstream genes. Among these genes, we confirmed that *HNF1B*, which encodes a protein responsible for ICM morphology and blastocoel formation (28), was affected by the miR-125b/SUV39H1 axis during EGA. Unsurprisingly, the depletion of *HNF1B* during NT reprogramming caused development defects similar to those observed with miR-125b deletion. We conclude that HNF1B may act downstream of miR-125b/SUV39H1 during NT-mediated reprogramming.

In summary, we clearly demonstrate that miR-125b regulates the developmental capacities of bovine preimplantation SCNT embryos and affects both histone modification and heterochromatin relaxation. Briefly, miR-125b directly represses the SUV39H1 expression and H3K9me3-initiated heterochromatin deposition, and these changes play prerequisite roles for enhanced EGA. Moreover, we identified a downstream gene *HNF1B* that may be required to mediate the effects of the miR-125b/SUV39H1 axis on SCNT embryonic development, thus providing a possible explanation for the observed differences in the development capacities of the embryos. Taken together, our results not only reveal an important regulatory role for the miR-125b/SUV39H1 interaction in SCNT embryonic development but also identify a previously unknown link between miRNA, histone methylation, and EGA during NT-mediated reprogramming.

## Experimental procedures

### Donor somatic cell culture, nuclear transfer, and in vitro reconstituted embryo culture

Ovaries were collected from a local abattoir, placed in sterile saline at 20 °C, and transported to the laboratory within 4–6 h. *In vitro* oocyte maturation, enucleation, microinjection, and reconstructed oocyte fusion were performed in our laboratory according to previously described methods (40). After activation, the activated embryos were cultured in drops of 50 μl of modified oviduct synthetic fluid with amino acids (mSOFaa) medium supplemented with 8 mg/ml BSA under a humidified atmosphere with 5% CO_2_ in air at 38.5 °C until use. BFFs were derived from approximately 30-day-old fetuses and cultured in Dulbecco's modified Eagle's medium supplemented with 10% fetal bovine serum. Cells at passage 3–5 were used as donor cells. Serum deprivation was used to induce the G_0_/G_1_ phase.

### Embryonic transfer of miRNA, siRNA, and mRNA

The miRNA mimic, miRNA inhibitor, and bovine SUV39H1-specific siRNA were diluted in nuclease-free water to final concentrations of 800 nm, 25 μm, and 16 μm, respectively. SCNT-generated embryos were injected with 10 pl of siRNA/miRNA at 8–9 h after activation (*i.e.* the pronuclear/one-cell stage).

SUV39H1, KDM4A, KDM4B, and GFP cDNAs were cloned into T7-driven vectors and synthesized using the mMESSAGE mMACHINE T7 Ultra Kit (Life Technologies, Grand Island, NY) according to the manufacturer's instructions. The storage concentration of each mRNA was optimized to 800 ng/μl. The integrity of the manufactured mRNA was confirmed by electrophoresis with formaldehyde gels. SCNT-generated embryos were injected with 10 pl of mRNA at 8–9 h after activation. GFP mRNA was used as a control.

### In vitro fertilization

A 50-μl sperm suspension (2 × 10^6^ spermatozoa/ml) was added to 20–25 cumulus–oocyte complexes (COCs) in a 50-μl microdrop of the BO-IVF medium (IVF Bioscience, Falmouth, UK) under mineral oil. After 16 h of *in vitro* fertilization, cumulus cells and redundant spermatozoa were dispersed from the zygotes with phosphate-buffered saline (PBS) supplemented with 0.1% bovine testicular hyaluronidase, and then the zygotes were transferred to be cultured in mSOFaa with 8 mg/ml BSA until use.

### RNA isolation, reverse transcription PCR (RT-PCR), and qPCR

Total RNA was isolated from SCNT embryos (*n* = 10 embryos/pool) using the Cells-to-Signal™ kit (Ambion Co., Austin, TX) according to the manufacturer's instructions. The M-MLV RT reagent included in the kit was used for reverse transcription. Quantitative PCR was performed on an ABI StepOnePlus PCR system (Applied Biosystems, Foster City, CA), and the results were normalized to GAPDH mRNA levels. Data are expressed as FC = 2^−ΔΔCt^. The primer sequences used for qPCR are listed in supplemental Table S1.

miRNA samples (*n* = 50 embryos/pool) were isolated using the miScript Single Cell qPCR Kit (Qiagen, Hilden, Germany) and reverse transcribed using a miScript II RT kit (Qiagen). Mature miRNA expression was quantified using a miScript SYBR Green PCR kit containing 10× miScript Universal Primer (Qiagen), according to the manufacturer's instructions. U6 was quantified and used as an internal control to normalize miRNA expression levels.

### RNA-seq and library construction

Twenty 8-cell embryos for control and the si-SUV39H1-injected group were directly lysed and used for cDNA synthesis using the SMARTer® v4 Ultra^TM^ Low RNA kit for Illumina® sequencing (Clontech), according to the manufacturer's protocol.

The quality-ensured RNA-seq libraries were pooled and sequenced on HiSeq 2500 platform with 100- or 150-bp–paired end modes (sequenced by Novogene). Sequencing reads were aligned to the bovine reference genome (UMD 3.1) with the Tophat software and fragments per kilobase of transcript per million mapped reads (FPKM) of each gene was calculated using Cufflinks. The RNA-seq data have been deposited in Gene Expression Omnibus under the accession number GSE99072.

### Immunoblot analysis

Embryos (*n* = 200 embryos per pool) were lysed in radioimmune precipitation assay buffer, and proteins were separated on 10–15% polyacrylamide gels at 150 V for 70–90 min. Subsequent PVDF membrane transfers were performed at 250 mA for 50 min at 4 °C. After the transfer procedure, membranes were incubated for 1 h in blocking buffer (Tris-buffered saline, pH 7.4 (TBS), with 0.1% (v/v) Tween 20 and 5% (w/v) nonfat milk at room temperature) followed by incubation overnight with one of the following primary antibodies in blocking buffer on a rotating shaker at 4 °C: anti-H3K9me3 (1:300 dilution; Abcam, Cambridge, UK) and anti-GAPDH (1:1000 dilution; TransGen Biotech, Beijing, China). The membranes were then washed and incubated with a horseradish peroxidase-conjugated anti-rabbit-IgG secondary antibody at (1:500 dilution; Thermo Scientific, Waltham, MA) for 1 h at room temperature. Immunoreactive proteins were visualized by autography with the SuperSignal West Pico Chemiluminescent substrate (Thermo Scientific, Rockford, IL). The protein concentrations of BFF lysates were measured using the bicinchoninic acid method. The protein separation, blotting, and visualization procedures were performed as described above.

### Immunofluorescence analysis

Zona pellucida-free embryos were washed 3 times in PBS, fixed in freshly prepared 4% paraformaldehyde in PBS, permeabilized in 1% Triton X-100 in PBS, and incubated in blocking solution (1% bovine serum albumin in PBS) for 1 h. For immunolabeling, the embryos were incubated overnight at 4 °C with an anti-H3K9me3 antibody (1:800 dilution; ab8898, Abcam) followed by 3 washes with PBS and a 1-h incubation with a FITC-labeled goat anti-rabbit IgG (1:1000 dilution; Beyotime, Shanghai, China) secondary antibody. The samples were then washed and counterstained with 4,6-diamidino-2-phenylindole (DAPI; Beyotime) to label DNA. Fluorescence was analyzed using a Nikon Eclipse Ti-S microscope equipped with a 198 Nikon DS-Ri1 digital camera (Nikon, Tokyo, Japan).

The cell numbers in blastocysts were estimated by counting nuclei stained using DAPI, and the number of TE cells was determined by counting nuclei positive for CDX2. The cell number of ICM was estimated as the total number of nuclei minus the number of TE nuclei (41, 42). The blastocyst was incubated with anti-CDX2 (1:200, BioGenex Inc., San Ramon, CA) for detecting the TE, and the secondary antibody was Alexa Fluor Cy3-labeled goat anti-mouse IgG (1:1000 dilution; Beyotime, Shanghai, China).

### Statistical analysis

The statistical analysis was performed using SPSS 13.0 for Windows (SPSS, Inc., Chicago, IL) and Excel (MicroSoft Corp., Redmond, WA). All data are presented as the means ± standard deviations and were analyzed using the two-tailed Student's *t* test for pairwise comparisons or an analysis of variance for multiple comparisons. A *p* < 0.05 (two-tailed) was considered statistically significant.

## Author contributions

Y. Z. and J. Z. designed and conceived the experiments. X. L., P. Q., Y. W., C. Z., and X. M. conducted the oocyte and embryo manipulations. J. Z. and M. W. conducted the molecular experiments. J. Z., J. L., and J. S. contributed to the bioinformatics analysis. J. Z. conducted the cell manipulations. Y. Z., Y. W., and J. Z. wrote the manuscript, and all authors reviewed the manuscript.

## Supplementary Material

Supplemental Data

## Footnotes

4 The abbreviations used are:

SCNT: somatic cell nuclear transfer
miRNA: microRNA
qPCR: quantitative real-time PCR
BFF: bovine fetal fibroblast
EGA: embryo genome activation
GO: Gene Ontology
NT: nuclear transfer
TE cells: trophectoderm cells
ICM: inner cell mass.
