# Supplementary material for: MicroRNA-125b is a key epigenetic regulatory factor that promotes nuclear transfer reprogramming
Source: J Biol Chem. 2017 Aug 9;292(38):15916–26. doi: 10.1074/jbc.M117.796771 (PMC5612121; doi:10.1074/jbc.M117.796771)
Supplement: Supplemental Data [file supp_292_38_15916__index.html]

microRNA-125b is a key epigenetic regulatory factor that promotes nuclear transfer reprogramming — MicroRNA-125b is a key epigenetic regulatory factor that promotes nuclear transfer reprogramming — miR-125b facilitates nuclear transfer reprogramming — Supplemental Data 

# MicroRNA-125b is a key epigenetic regulatory factor that promotes nuclear transfer reprogramming

## Supplemental Data

- Supplemental Data (.docx, 2.9 MB) - Supplemental Tables and Figures
