# Supplementary material for: MicroRNA-125b is a key epigenetic regulatory factor that promotes nuclear transfer reprogramming
Source: J Biol Chem. 2017 Aug 9;292(38):15916–26. doi: 10.1074/jbc.M117.796771 (PMC5612121; doi:10.1074/jbc.M117.796771)
Supplement: Supplemental Data [file 10.1074_M117.796771_jbc.M117.796771-1.docx]

**microRNA-125b is a key epigenetic regulatory factor that promotes nuclear transfer reprogramming**

Jingcheng Zhang^a†^, Pengxiang Qu^a†^, Chuan Zhou^a^, Xin Liu^a^, Xiaonan Ma^a^, Mengyun Wang^a^, Yongsheng Wang^a,1^, Jianmin Su^a^, Jun Liu^a^, Yong Zhang^a,1^

*^a^ Key Laboratory of Animal Biotechnology of the Ministry of Agriculture, College of Veterinary Medicine, Northwest A&F University, Yangling 712100, Shaanxi, China.*

Supplemental Table S1

Supplemental Figure S1

Supplemental Figure S2

Supplemental Figure S3

Supplemental Figure S4

Supplemental Figure S5

Supplemental Figure S6

**Table S1. Primer information**

| Name | Sequence（5‘-3’） |
| --- | --- |
| qbta-miR-125b F* | GCGGTCCCTGAGACCCTAAC |
| U6 F* | CGCTTCGGCAGCACATATAC |
| autosomal satellite F | TGGAAGCAAAGAACCCCGCT |
| autosomal satellite R | TCGTGAGAAACCGCACACTG |
| SUV39H1 F | CATAGACAACCTTGATGAGCG |
| SUV39H1 R | GCAGGATTCAGTCCCACAC |
| GAPDH F | CTGGAGAAACCTGCCAAGTATG |
| GAPDH R | GAGTGTCGCTGTTGAAGTCGC |
| HNF1B F | CTGCAGTGACGCAGCTACAGAAC |
| HNF1B R | ACCACCATCGCAGATGGAAAC |
| LIN28 F | AGACAGGTGCTACAACTGTGGAG |
| LIN28 R | GGCAGAGCTATGGATCTCTTCTT |
| SEBOX F | CCCACCCTTATTCCCACGA |
| SEBOX R | GCAGAGTGAGGAGCCAGACA |
| KLF4 F | CTGCTCACGACTTTCCCTTG |
| KLF4 R | GCAAACTTCCACCCACAACC |
| RPL23 F | CTGACAACACAGGAGCCAAAA |
| RPL23 R | ATCACCATGTCACCCACACC |
| CDC2 F | GGGTCAGCTGGCTACTCAAC |
| CDC2 R | AGTGCCCAAAGCTCTGAAAA |
| HSPA1A F | GGGGAGGACTTCGACAACAGG |
| HSPA1A R | CGGAACAGGTCGGAGCACAGC |
| UBE2A F | GGGCTCCGTCTGAGAACAACATC |
| UBE2A R | CATACTCCCGCTTGTTCTCCTGG |
| HOXB4 F | GGCGACCATTACCTCGACA |
| HOXB4 R | CAGGAATGAGGGACAAAGAAAGA |
| UBTFL1 F | ATGAAGGAGGGAAACAGACACC |
| UBTFL1 R | GGAGAAGAGCACACACAGGTAAAA |
| SLC8A2 F | ACTAACAGCCTTCACTCTT |
| SLC8A2 R | GAACATAGACAGTAACGAACA |
| ZSCAN5B F | AGGTTTGGCTTTGTATCAGTGGTT |
| ZSCAN5B R | GGCACAGGTCTCTCTCATCGT |
| DDB2 F | TGGCATAAAGGACAAACCTACCT |
| DDB2 R | GACCACCACTCGGCTTTTG |
| CXCL6 F | CCACCACACCGGGAATTC |
| CXCL6 R | TCCCGCGGCGATCAC |
| KDM4A F | GTGTTCAGCCAATGCCCTGGAA |
| KDM4A R | AGCCTTACCACCTCACACTGGT |
| KDM4B F | CCGTGAAGTGTGGCCATCCCT |
| KDM4B R | ACGTCTGCCGTGGCTTCCAC |
| KDM4D F | AGTTGGCAGATGTGGTTCTC |
| KDM4D R | GATTGTCCATCCGTCCCTC |

* The reverse primer for qPCR experiments of miRNAs is a universal primer provided by the miScript II RT Kit (QIAGEN).


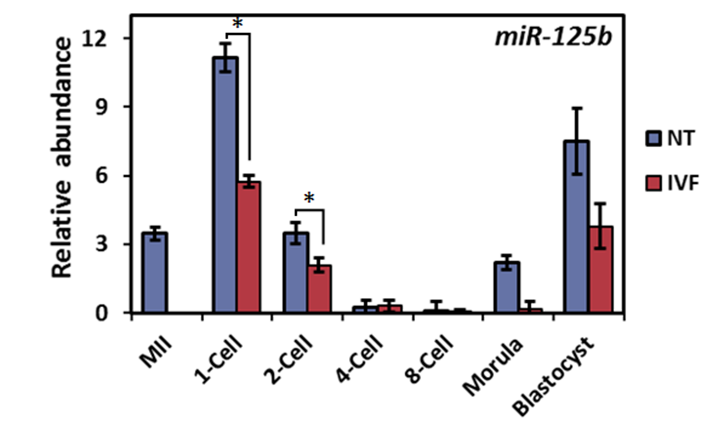


**SUPPLEMENTAL FIGURE S1.** qRT-PCR results showing the relative abundance of miR-125b in IVF/ NT-embryos. Values shown are normalized to U6 expression. Error bars indicate the SEM.

**
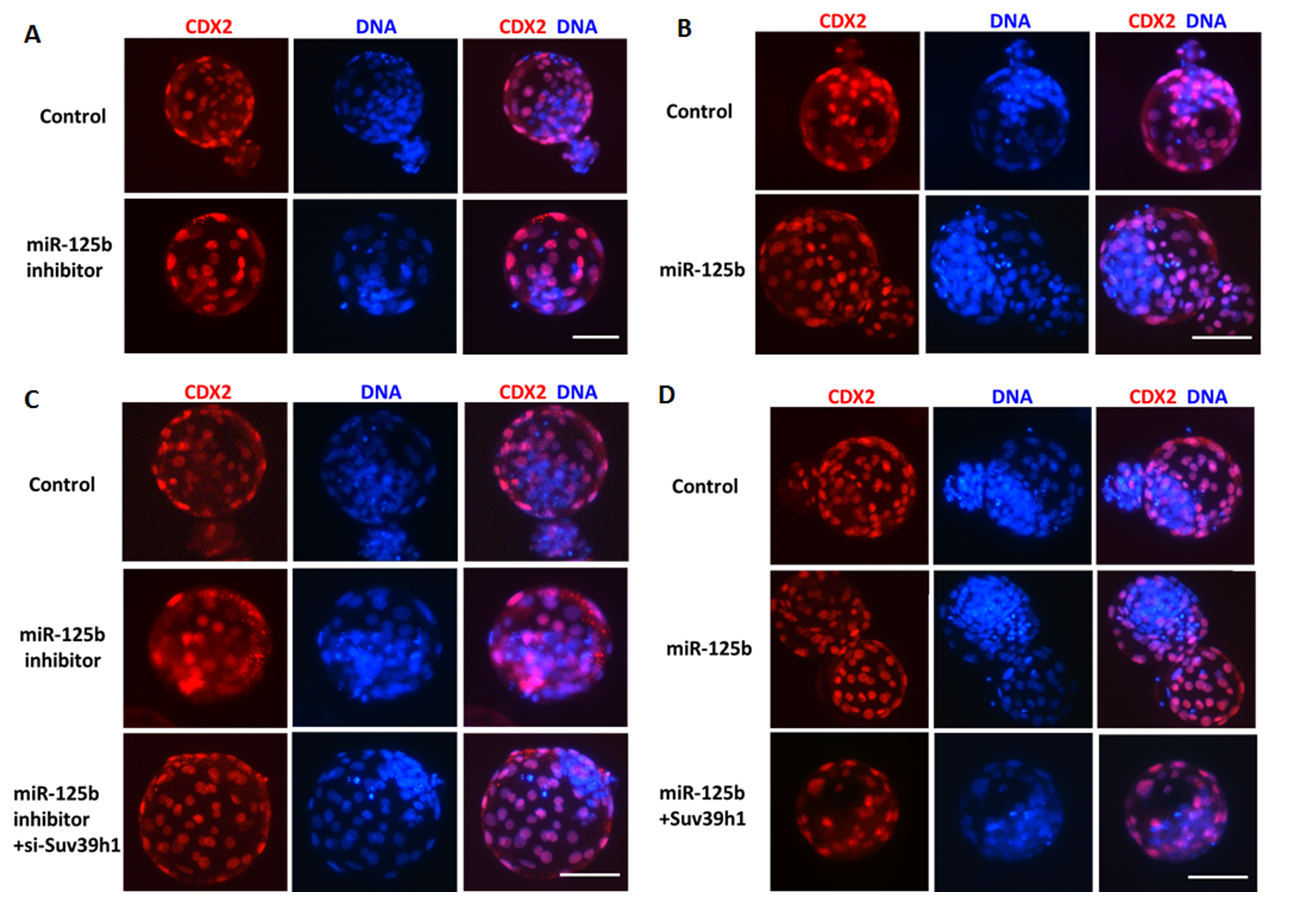
**

**SUPPLEMENTAL FIGURE S2.** Representative images of labeling of inner cell mass and trophectoderm cells. Blastocysts were harvested at day 8 and subjected to differential immunofluorescence. Blue: DAPI staining (all nuclei); red: CDX2^+^ cells (labeled with mouse anti-CDX2 and Cy3 anti mouse IgG). Trophectoderm cells were labeled with DAPI and anti CDX2 while inner cell mass cells were labeled with DAPI only. Related to Figures 1 and 5.


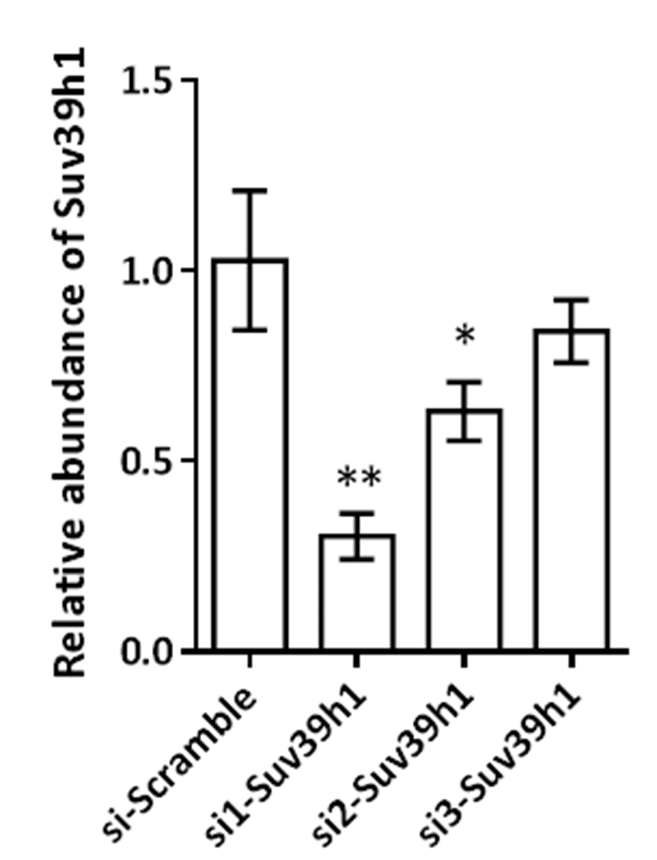


**SUPPLEMENTAL FIGURE S3.** qPCR analysis of si-Suv39h1 knockdown efficiency. 1-Cell SCNT embryos were injected with Suv39h1 siRNA or negative control siRNA. Embryos were then sampled at 44 hpi (n = 3 pools of 12 embryos each per treatment) for qPCR analysis. Data shown are mean expression values relative to GAPDH. Efficiency Error bars indicate SEM.


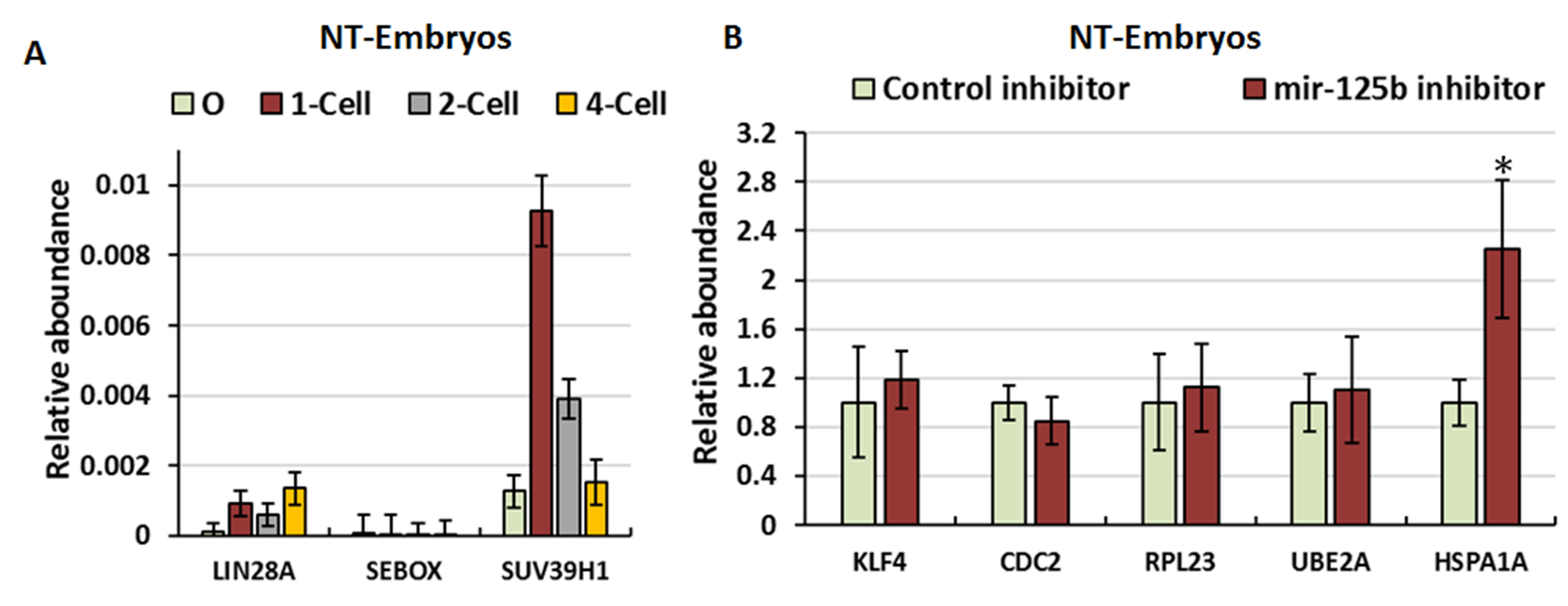


**SUPPLEMENTAL FIGURE S4.** A. qPCR analysis the target-gene abundance of miR-125 during the early stage of SCNT embryos. B. qPCR analysis the downstream genes of miR-125/LIN28/SEBOX pathway during the EGA stage of SCNT embryos.


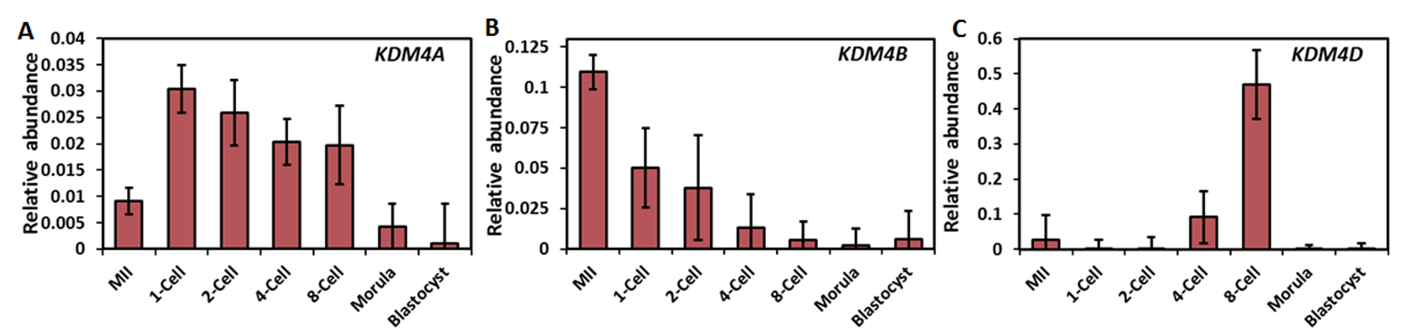


**SUPPLEMENTAL FIGURE S5.** qPCR analysis the relative abundance of KDM4A(A), KDM4B(B) and KDM4D(C) during the early stage of SCNT embryos. The GAPDH expression level was set to 1.0. Bars indicate the SEM.

**
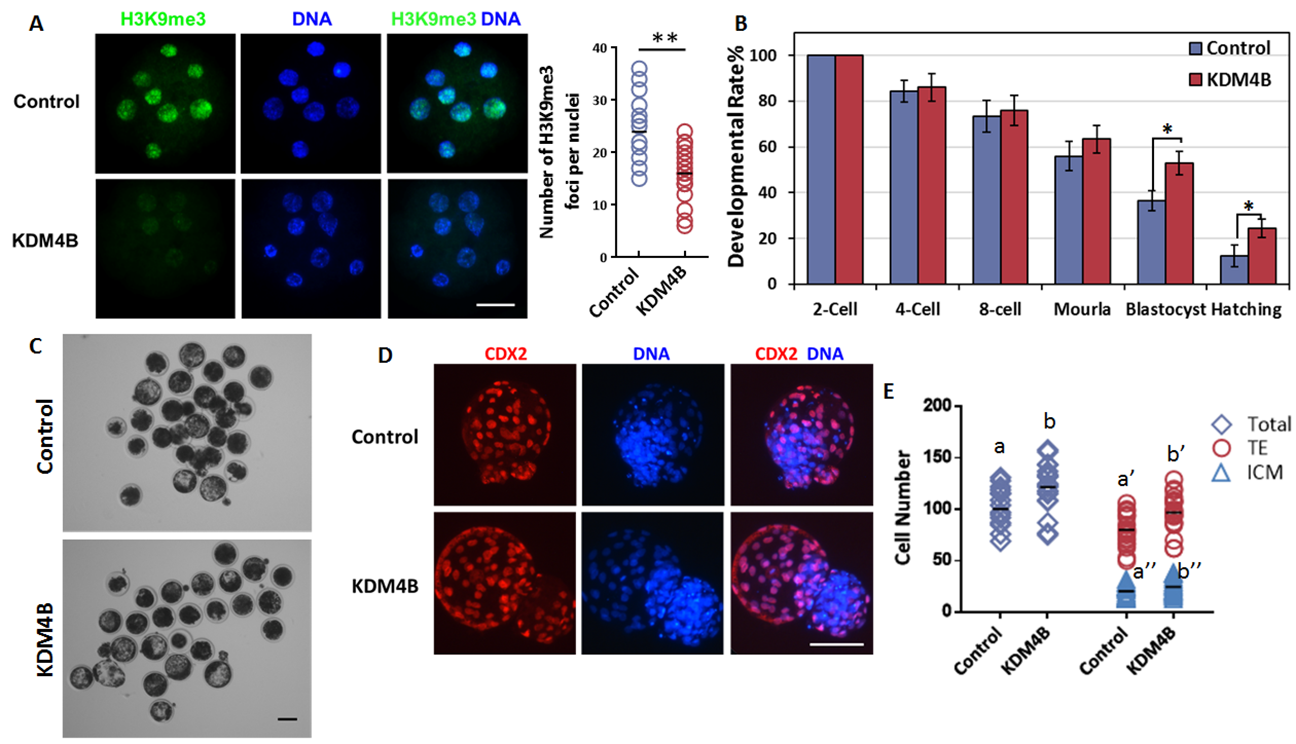
**

**SUPPLEMENTAL FIGURE S6.** Injection of bovine KDM4B mRNA improves developmental potential of bovine SCNT embryos.

1. Injection of KDM4B can reduce H3K9me3 mark in 8-cell SCNT embryos. Scale bars = 100 μm
2. KDM4B mRNA injection improves development of bovine SCNT embryos. Shown is the percentage of embryos that reached the indicated stages. Error bars indicate SD.
3. Representative images of control and KDM4B-injected SCNT-embryos at 8 days post-activation. Scale bar = 150 μm.
4. KDM4B mRNA injection improves SCNT-blastocyst quality. Immunostaining of CDX2 (red) and DAPI (blue) in blastocysts developed from control and KDM4B injeced. Scale bars = 100 μm.
5. The plot graph shows the total; ICM and TE cell numbers of control and KDM4B-injected somatic cell NT-embryos. Different characters a, b (total), a’, b’ (TE), or a’’, b’’ (ICM) above plots indicate significant differences (P < 0.05).
